# Demulsification of crude oil-in-water emulsions by means of fungal spores

**DOI:** 10.1371/journal.pone.0170985

**Published:** 2017-02-24

**Authors:** Alba Adriana Vallejo-Cardona, Rafael Martínez-Palou, Benjamín Chávez-Gómez, Graciela García-Caloca, Jairo Guerra-Camacho, Ricardo Cerón-Camacho, Jesús Reyes-Ávila, James Robert Karamath, Jorge Aburto

**Affiliations:** 1 CONACYT-Centro de Investigación y Asistencia en Tecnología y Diseño del Estado de Jalisco, A.C., Av. Normalistas 800, Colinas de la Normal, C.P., Guadalajara, Jalisco, México; 2 Dirección de Investigación en Transformación de Hidrocarburos. Gerencia de Transformación de Biomasa. Instituto Mexicano del Petróleo, Eje Central Lázaro Cárdenas Norte 152, CP, Mexico City, Mexico; MEXICO

## Abstract

The present feature describes for the first time the application of spores from *Aspergillus sp*. IMPMS7 to break out crude oil-in-water emulsions (O/W). The fungal spores were isolated from marine sediments polluted with petroleum hydrocarbons. The spores exhibited the ability to destabilize different O/W emulsions prepared with medium, heavy or extra-heavy Mexican crude oils with specific gravities between 10.1 and 21.2°API. The isolated fungal spores showed a high hydrophobic power of 89.3 ± 1.9% and with 2 g of spores per liter of emulsion, the half-life for emulsion destabilization was roughly 3.5 and 0.7 h for extra-heavy and medium crude oil, respectively. Then, the kinetics of water separation and the breaking of the O/W emulsion prepared with heavy oil through a spectrofluorometric technique were studied. A decrease in the fluorescence ratio at 339 and 326 nm (I339/I326) was observed in emulsions treated with spores, which is similar to previously reported results using chemical demulsifiers.

## Introduction

Mature petroleum reservoirs produce crude oil with large quantities of water due to the employment of enhanced recovery methods that require the use of high amount of water. Most of such water is emulsified into crude oil during production, which increments the viscosity and makes flow more difficult which causing operational issues concerning production, transportation and refining that impact company productivity.

The kind of emulsions present during petroleum production is quite complex but, they are mainly formed by water-in-oil-emulsion (W/O) because natural demulsifiers present in crude oil such as asphaltenes, resins and napthenic acids favor the formation of such emulsions while others may be present in considerably less proportions, i.e. oil-in-water (O/W), water-in-oil-in-water (W/O/W), etc.

In recent years, the deliberate emulsifier-assisted formation of heavy crude oil-in-water (O/W) emulsions has been used as a technical strategy to reduce the viscosity of heavy oils to facilitate their transportation [1, 2].

Nevertheless, after oil has been transported to the refinery and before being processed, the O/W emulsions must be broken in order to remove water and prevent corrosion problems in pipes and equipment from happening [3].

There are several physical (thermal, mechanical, electrical) and chemical (addition of demulsifiers) methods currently used to break crude oil (O/W) emulsions and dehydrate crude oil [4], however microbiological methods aimed at accomplishing this goal are scarce to date.

Chemical demulsifiers is still the most widely employed method to break crude oil emulsions but in many cases these demulsifiers are toxic and generate environmental problem and can affect the health of operating personnel [5].

Many biodemulsifiers-producing bacteria have been isolated and studied such as *B*. *subtilis* [6], *Alcaligenes sp*. [7] and *B*. *mojaventis XH1* [8], Micrococcus species [9] and mixed bacterial cultures [10], but in most of the cases, they have been tested in the breaking of model W/O emulsions.

Park *et al*. observed that a suspension of spores of *Streptomyces sp*. exerted a strong effect on the stability of emulsions made from solvents, oils and commercial surfactants. Such spores presented a hydrophobic character, which varies with the culture medium used during growing [11]. The ability to break O/W emulsions from heavy crude oils is based on the hydrophobicity of these spores. To the best of our knowledge, no studies of de-emulsification with spore have been reported for emulsions obtained from extra-heavy oils.

Coutinho et al. investigated in 2013 the demulsifying properties of extracellular products and cells of *Pseudomonas aeruginosa* MSJ isolated from petroleum-contaminated soil for W/O or O/W and industrial emulsions when cultivated in media with different carbon sources. The addition of cells and supernatants of cultures of *P*. *aeruginosa* MSJ promoted breakage of W/O and O/W emulsions consisting of different organic phases and emulsifying agents [12].

Recently, a highly efficient demulsifiying strain, LH-6, isolated from petroleum contaminated soil was investigated for the demulsification of O/W and W/O model emulsions. By using the optimized cultivation conditions, demulsification efficiencies higher than 97% were obtained [13]. However, in this work and in most of the studies with biodemulsifiers, model emulsions with very low viscosity and with low stability and consequently, easy to break are used. In most cases, these emulsions break spontaneously after hours of having been formed without requiring demulsifiers; however, natural emulsions obtained from heavy and extra heavy crude oils are much more complex, stable and difficult to break and breaking through an environmentally friendly method remains a major challenge.

In this work, the effect of *Aspergillus sp*. IMPMS7 spores isolated from marine sediments was studied on the breaking of Mexican medium, heavy and extra-heavy crude oil-in-water (70:30) emulsions was studied. The spores of *Aspergillus sp*. IMPMS7 showed a good performance to break the O/W emulsions with high hydrophobic power of 89.3 ± 1.9%. With 2 g of spores per liter of emulsion, the half-life for emulsion destabilization was roughly 6.12, 4.4 and 1.06 h for extra-heavy, heavy and medium crude oil, respectively. The spectrofluorometric technique was employed as a useful tool to study the process. A decrease in the fluorescence ratio at 339 and 326 nm (I339/I326) was observed in emulsions prepared with heavy oil.

## Materials and methods

### Isolation of microorganisms

Microorganisms were isolated from 28 samples of marine sediments from the Gulf of Mexico. The samples were kept under refrigeration. Each sediment sample was inoculated in a Petri dish medium containing 0.4 g KH_2_PO_4_, 1.6 g K_2_HPO_4_, 1.5 g NH_4_Cl, 0.17 g MgCl_2_-6H_2_O, 0.15 g Na_2_SO_4_.7H_2_O, 0.045 g CaCl_2_-2H_2_O, and 15 g agar powder in a modified mineral solution (1 L). The composition of the modified mineral solution was: 5.1 g/L of MgCl_2_.2H_2_O, 0.66 g of MnCl_2_.H_2_O, 1 g of NaCl, 1 g of FeCl_3_.6H_2_O, 0.1g of CaCl_2_.2H_2_O, 0.01 g CuCl_2_, 0.08 g of ZnCl_2_, 0.05 g of AlCl_3_, 0.01g of H_3_BO_3_ and 0.04 g of Na_2_MoO_4_.2H_2_O. The surfactant and carbon source was Ethoxylate Nonylphenol (ENP, ethoxy groups 15 mol, HLB = 15.0) at 1000 ppm in water. This kind of compound can be efficiently degraded by *Aspergillus sp*. [14]. The Petri dishes were incubated at 35°C for 4 days and the resulting colonies were isolated and subsequently tested for their ability to destabilize O/W emulsions formulated with medium crude oil.

### Production of spores

The isolated fungus was seeded in Petri dishes which contained the culture medium adjusted to a pH of 8.0 ± 0.2 for the propagation of spores with the following composition: glucose, 20 g/L; NH_4_Cl, 2 g/L; KH_2_PO_4_, 1 g/L; K_2_HPO_4_ 1 g/L; yeast extract, 0.5 g/L; agar 20 g and 1000 mL of synthetic seawater. The Petri dishes were incubated for 4 days at 35°C. The spores were harvested directly to determine their humidity (67.5%) and subsequently tested for demulsification of O/W emulsions.

### Identification of fungi

Phenotypic characteristics, i.e. colony morphology, color and growth rate on prepared slides were studied by means of microscopic observations. The potato dextrose agar (PDA) is used for the cultivation of fungi. Isolated spores were examined to complement the phylogenetic analysis using the taxonomic for *Aspergillus* [15].

DNA extraction, D1/D2 26S rRNA PCR amplification and sequencing were performed as previously reported [16]. The sequences were subjected to a BLAST (www.blast.com) to search for the taxonomic hierarchy of the sequences. A collection of taxonomically related fungal sequences were obtained from the NCBI Taxonomy Homepage (http://www.ncbi.nlm.nih.gov/Taxonomy). CLUSTAL X program was used to perform a multiple alignment analysis [17] in the SEAVIEW software [18]. The neighbor-joining phylogenetic tree with 1,000 bootstrap replications [19] was constructed in the MEGA 5.05 program [20].

### Characterization of crude oils

The crude oil samples used in this study were provided by the Mexican Petroleum Company (PEMEX) from off- and on-shore reservoirs from a marine well drilled in the south of the Gulf of Mexico (18.776471, -91.766473) and were characterized by the following standard procedures: the samples were characterized by API gravity (ASTM D-287), kinematic viscosity (ASTM D-445), salt content (ASTM-D-3230), paraffin content (UOP-46), water content (ASTM D-4006), and saturated, aromatic, resin and asphaltene content (ASTM D-2007). Total sulfur was determined in 9000S Sulfur Analyzer from ATEK instruments (http://www.speciation.net/Database/Instruments/Antek/MODEL-9000-NitrogenSulfur-Analyzer-;i2248), employing the standard procedure ASTM D 5453–05.

### Preparation of O/W emulsions

To obtain the emulsions, synthetic seawater was prepared according to the corresponding standard method [20], taking into account the original water content of the crude oil. The utilized NPE surfactant containing 15 mol of ethoxy group, is a commercially available surfactant, a white waxy solid with HLB of 15.0. NPE was first dissolved in the synthetic seawater and the resultant solution was poured into a jacketed glass reactor with water recirculation at 25°C as described previously [2, 3]. The crude oil sample was added to obtain a water content ratio in the O/W emulsion of 30% w/w. The reactor was incubated at 25°C for ten minutes and the emulsions were mixed using an IKA Labortechnik homogenizer for 5 min at 8000 rpm. The formation of the O/W emulsion was corroborated by dispersing an emulsion drop in water and observing with optical microscopy and characterizing them Differential Scanning Calorimetry (DSC). The O/W emulsions prepared in this way for the three crude oils were stable during two weeks at room temperature without phase separation.

### Determination of the content of water separated from the emulsions

The kinetic of the emulsion breaking was followed by the water separated from the emulsion after the addition of the spores. The separation of water (SW, %) from the fresh and treated emulsions was determined according to Eq 1. The content of water remaining in the crude oil (*V*_*KF*_) was determined by the Karl Fischer potentiometric method, in accordance with procedure ASTM D1744.

$$SW\;(vol.\%)=\frac{V^{{}^{\circ}}-V_{KF}}{V^{{}^{\circ}}}\times 100$$ (1)

Where *V*° is the original volume of water in the emulsion.

### Test for the demulsification of O/W emulsions

Different spore concentrations (0.5 to 5 g/L) were added to each freshly prepared O/W emulsion. The spores were softly homogenized in the O/W emulsions by mechanical stirring for 1.5 minutes at 200 rpm. Each emulsion was incubated at 45°C and the separation of water was followed as a function of time. The O/W and spore treated emulsions were observed with a Nikon Eclipse E800 microscope equipped with xenon lamp.

### Hydrophobicity test of the spores

The hydrophobicity of the spores was determined by adapting the method proposed by Rosemberg et al. [21], which consists of a suspension of spores in 5 mL of a phosphate buffer at pH 7.0. The turbidity of the suspension was adjusted to approximately one unit of absorbance as read at 600 nm. Later, 2 mL of xylene were added to the tube containing the suspension, stirred in a Vortex stirrer for one minute and left to stand at room temperature. The spores migrated toward the organic phase, changing the observed turbidity of the system. The hydrophobicity percentage was obtained from Eq 2:

$$Spore\;hydrophobicity\;\left(\%\right)=\left(1-\frac{OD_{t=20min}}{OD_{t=0min}}\right)\times 100$$ (2)

in which OD_(t = 0 min)_ and OD_(t = 20 min)_ are the optical densities or turbidities of the suspension of the aqueous phase at 0 and 20 minutes after xylene addition.

Hydrophobicity is the physical property of a molecule or species (known as a hydrophobe) that is seemingly repelled from a mass of water. The hydrophobic power is a measure of how hydrophobic are the spores which is related to their ability to break the emulsion.

### Fluorometric spectroscopy

These measurements were performed on a RF-5301PC Shimadzu spectrofluorometer equipped with a 150 W Xenon lamp and a cell temperature controller. The emission spectra of the pure crude oil, the O/W emulsion and the treated emulsions were recorded between 298 and 421 nm using an excitation wavelength (λexc) of 290 nm. The temperature was maintained at 25°C and the spectra were recorded with a slit width of 3 nm. The scanning rate was fixed at 400 nm/min. Each crude oil emulsion (CO) was dissolved in a NPE solution (this concentration, 200 ppm, is up on of the CMC and keep of emulsion stability) and a corresponding aliquot was measured to prepare aqueous dispersions before performing the fluorometric measurements. The spectral center of mass (SCM, Eq 3) was calculated from the emission spectra as described previously [22]. The spectral center of mass (SCM) was determined from each spectrum (Eq 3), where I(λ) is the intensity of the fluorescence as a function the emission wavelength (λ).

$$SCM=\frac{\sum \lambda \cdot I(\lambda )}{\sum I(\lambda )}$$ (3)

The SCM was calculated for each CO concentration (incremented from 0 to 100 ppm) using the corresponding emission spectra. The value of the aggregation point (AP) was calculated from the point of intersection between the SCM curve as a function of the CO concentration and its first derivative. Determination of the SCM and AP permits the study of the CO in its microenvironment and how it and the interface vary with changes in CO concentration and the presence of NPE.

The SCM curve as a function of the concentration follows a hyperbola with two free parameters (Eq 4); the maximum change in the emission spectrum (SCM_max_) and the constant of dissociation constant of aggregation (K_S_^aggr^) related to the rapid equilibrium between the low and high states of aggregation of the crude oil molecules (Eq 5). [CO] and [CO-CO] represent the concentration of the crude oil in its dissociated and aggregated state respectively. This provides a simple criterion for the clustering degree of crude oil molecules within the concentration range used in this work. A similar approach permitted the estimation of the dimerization constants by fluorescence of the aromatic compound 4,6-dimethyl dibenzothiophene in solution [23]. In this case, the behavior can be associated with the dissociation or aggregation of the hydrocarbon molecules.

$$SCM=\frac{SCM_{\max}\cdot [CO]}{K_{S}^{aggr}+[CO]}$$ (4)

$$K_{S}^{aggr}=\frac{\left[CO]+[CO\right]}{\left[CO-CO\right]}$$ (5)

The ratio of intensities at 340 and 365 nm (IR = (I_340_/I_365_)) for every emission spectra were also calculated in order to study the microenvironment around the heavy crude oil (HCO) molecules and how this varies upon the addition of the spores. Fixed wavelength data as well as emission spectra were analyzed by means of the Panorama software. Appropriate blanks were employed to correct measurements for any light scattering contribution. By following excitation at 290 nm, the emission intensity at 340 nm was measured as a function of time.

## Results and discussion

### Characterization of crude oils

Samples of medium, heavy and extra-heavy (MCO, HCO, EHCO) crude oils employed in this study were characterized and the results are summarized in Table 1.

**Table 1:** Characterization of Mexican crude oils utilized in this study.

Parameter	Medium oil	Heavy oil	Extra heavy oil
API gravity (°)	21.2	16.4	10.1
Cinematic Viscosity (50°C, mm2/s)	292.8	505.1	2292.4
Salt content (lb/1000 bbl)	3.4	3.9	1245
Water content (%)	0.1	0.05	3.85
Total sulfur (%)	3.34	4.4	4.30
Total nitrogen (%)	0.27	0.29	0.37
Saturated	27.5	32.5	3.32
Aromatics	35.7	21.8	5.43
Resins	19.5	31.3	72.23
Asphaltenes	17.25	14.3	18.84

As it can be seen in Table 1, the three oils are viscous crude oils and with high gravity, especially heavy and extra-heavy crude oils samples. In general these actual samples contain many chemical compounds and high content of natural emulsifiers (i.e. asphaltenes and resins), in addition to the chemical emulsifier (NPE) added to prepare the emulsion, gave a very complex and stable emulsions and consequently difficult to break, in comparison with model emulsions obtained from kerosene and water commonly used in similar studies [11].

### Screening of spores as demulsifiers of O/W emulsions

**Fig 1:**
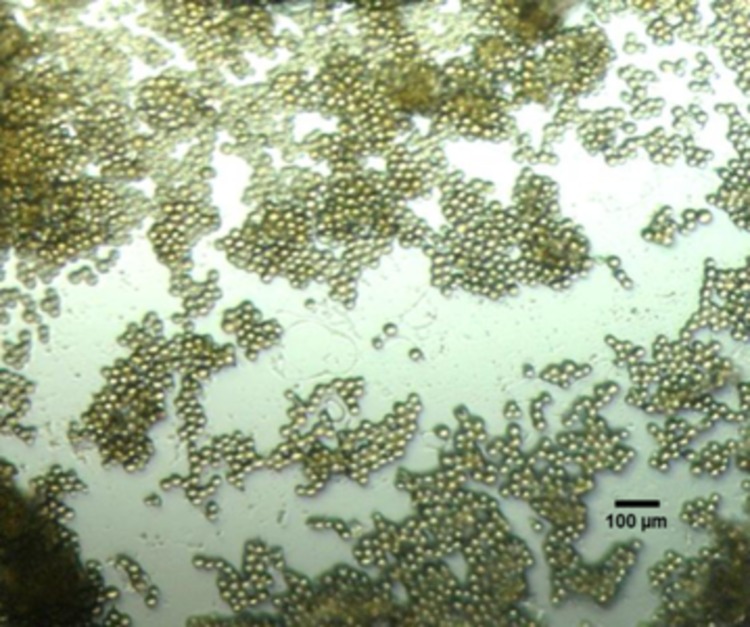
Optical microscopy of a spore suspension of the fungus *Aspergillus* sp. IMPMS7. Magnification 20x.

A total of 35 microorganisms including bacteria and fungi were isolated from marine sediments contaminated with petroleum hydrocarbons. Spores of *Aspergillus sp*. IMPMS7 were selected due to their ability to break the emulsion obtained from a heavy crude oil (21° API). Fig 1 shows a view of the spores imaged by an optical microscope.

### Fungus identification and determination of spore hydrophobicity

**Fig 2:**
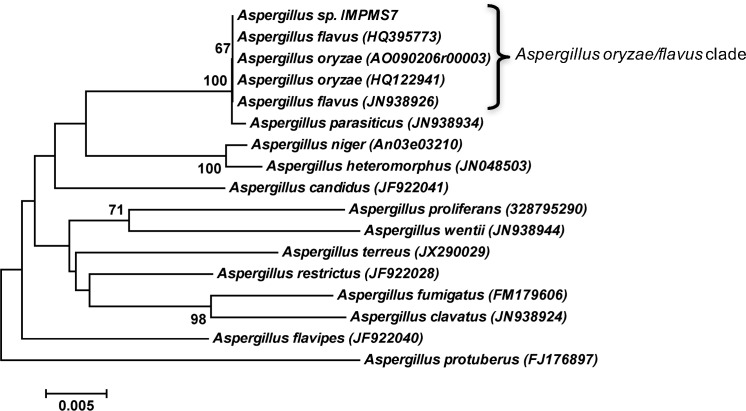
Neighbor-joining phylogenetic tree of *Aspergillus* species based on D1/D2 26S rRNA gene.

The isolated fungus was identified by means of a phylogenetic approach as a member of the *Aspergillus oryzae/flavus* clede (Fig 2). The scale bars indicate the nucleotide substitutions per site. Bootstrap values, expressed as the percentage of 1,000 replications, are given at the branching points; only values >50% are shown. The strain, named *Aspergillus sp*. IMPMS7, exhibited a similitude of 100% with all the members of *Aspertillus oryzae/flavus*. This fungus had the ability to degrade the surfactant nonylphenol ethoxylate (carbon source). To the best of our knowledge, this is the first time in which fungal spores are utilized as demulsifiers of O/W emulsions obtained from actual heavy and extra-heavy crude oils.

With respect to the spore hydrophobicity, a hydrophobicity value for *Aspergillus* spores of 89.3 ± 1.9% was obtained, which remained fairly constant for 9 days. A previous work reported the use of *Streptomyces* spores to destabilize petroleum distillate emulsions which presented a maximum hydrophobicity of 72% after 15 days [11].

### Characterization of emulsions

Emulsions obtained from extra-heavy crude oil, and heavy crude oil were characterized as previously reported [2, 3], where TQA employed in these works to prepare the emulsion is the same emulsifier than NPE used in this work. The corresponding characterization by means DSC for O/W emulsion using medium crude oil was performed under nitrogen atmosphere with a flow rate of 20 mL/min, using an aluminum pan. Three cycles from 50 to -60°C at a 10°C/min rate were used.

**Fig 3:**
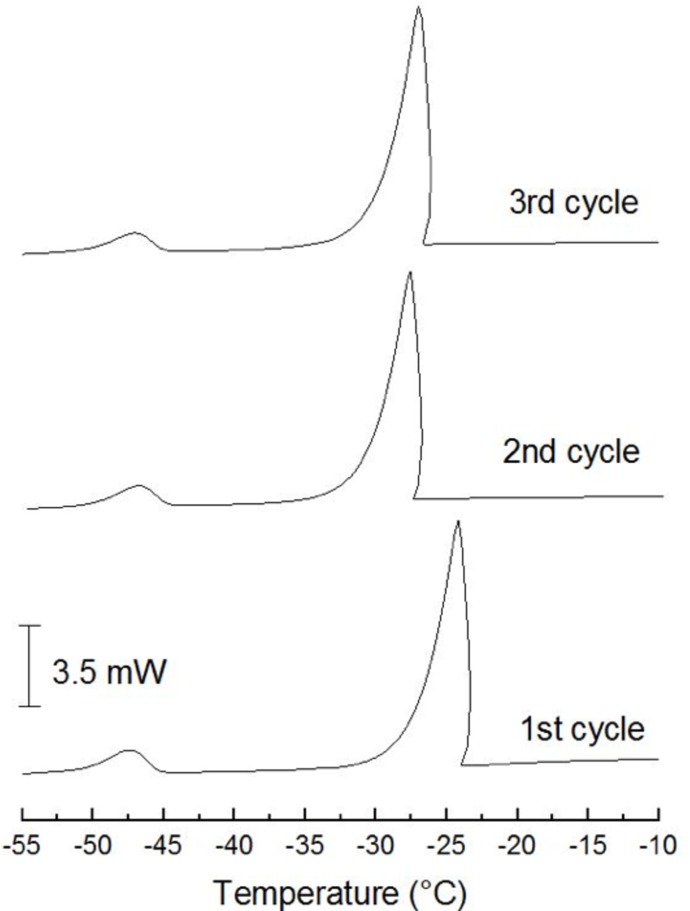
DSC thermograms for O/W emulsion with medium crude oil. Only the frozen cycle that give information about continuous phase are showed.

In all cases, O/W emulsions were obtained. In the Fig 3 it can be observed that in the first frozen cycle, an exothermic signal appears around -24°C which corresponds to the crystallization of water in the continuous phase; additionally is observed in -47°C a very low crystallization signal of micro-droplets into oil phase [24]. However, this represent low than 10%, for this reason we can consider as O/W emulsion.

**Fig 4:**
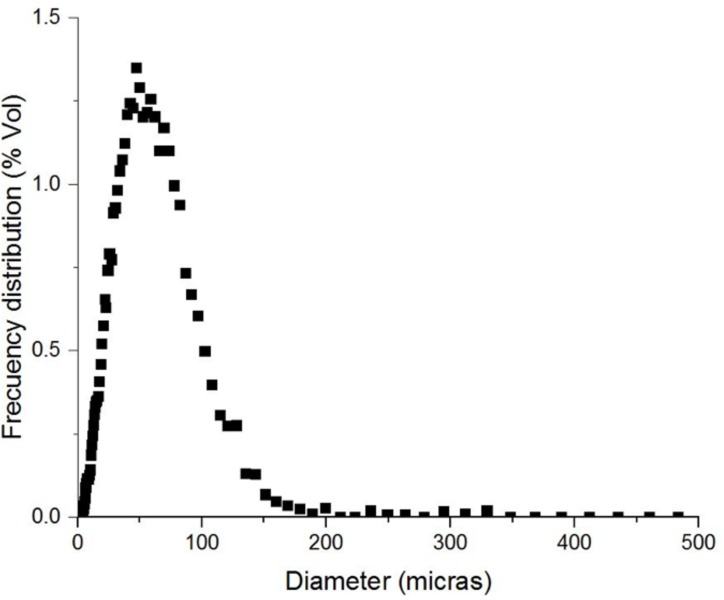
Particle size distribution for O/W emulsion with medium crude oil.

In Fig 4 observed de particle distribution of the same emulsion is showed. The particle size is fairly homogeneous and the mean correspond to 50 μm.

### Effect of the fungal spore concentration on the breakage of O/W emulsions

**Fig 5:**
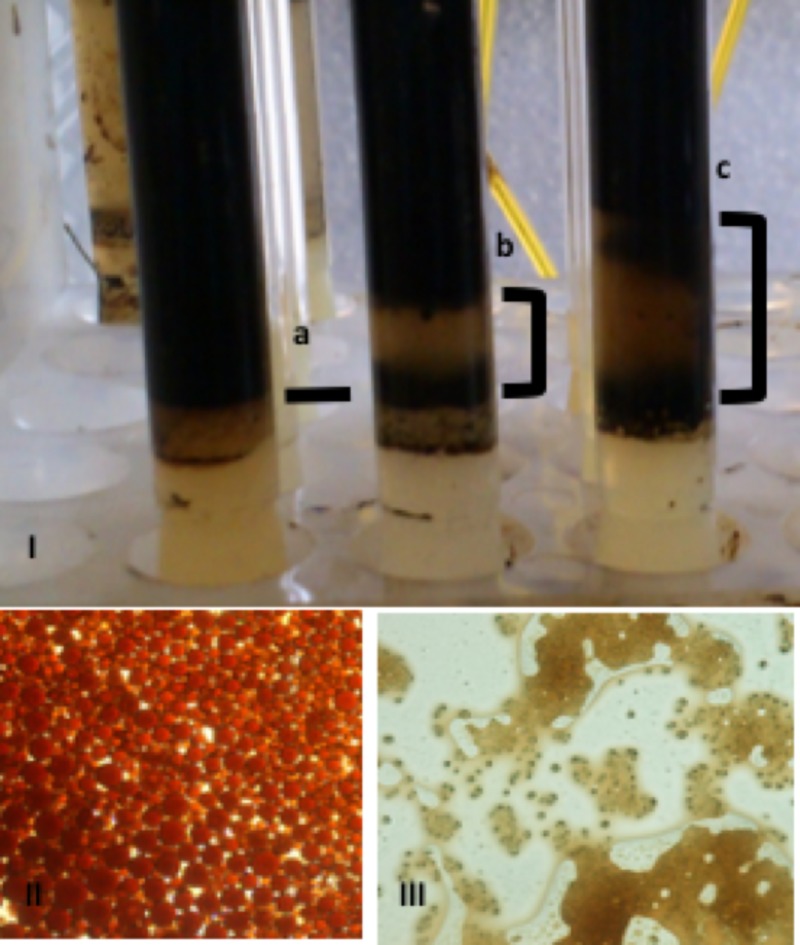
Photographs showing separation of water in the O/W emulsions prepared I) (a) without fungal spore, with fungal spores at a concentration of (b) 2 and (c) 3.5 g/L. Conditions: T = 45°C, 24 h of incubation. II) Micrograph of emulsion. and III) Micrograph after breaking of emulsion by spores. 20x.

Afterwards, the evaluation of the spore concentration on the emulsion breaking was performed by using 2.0 and 3.5 grams of spores per liter of emulsion (Fig 5). The brackets indicate the height of the column of water separated and it was reproducible after three repetitions of the experiments. The results were consistent with the decrease in water content in these crude detected by Karl-Fisher. The fungus spores were freshly harvested from the Petri dishes in which they were grown and added to O/W emulsions prepared with fresh crude oils of different specific gravities. The microscopic images were taken after emulsion preparation and after emulsion breaking by spores.

Previous studies determined the effect of bacterial biomass concentration on the breaking of kerosene-based O/W emulsions. Indeed, Das found that using inoculum concentrations of 10 and 15% v/v (bacterial *Microccus* counts: 5x10^8^ CFU ml^-1^), the bacterium *Microccus* decreased the demulsification half-time to one hour [9]. Recently, Mohebali and coworkers determined that using the bacterium *Ochrobactrum anthropi* at 3 g/L, and a maximum demulsification of 70% was achieved [25].

**Fig 6:**
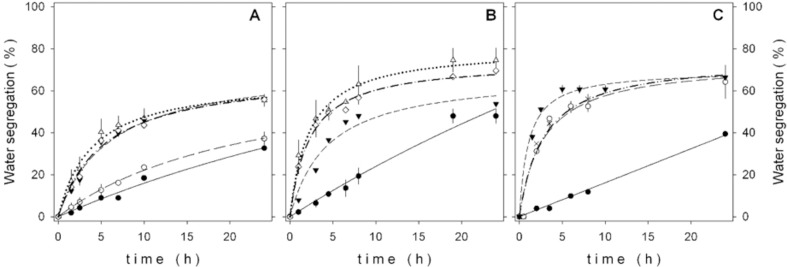
Effect of time on separation of water of O/W emulsions at 25°C with: medium(●, —), (▲, –––) heavy and extra heavy crude (▼, –⋅–) at 2.0 g/L spore concentration. The continue, discontinue and discontinue-point line represent hyperbolic curves obtained from least squares fitting. The vertical grey discontinue lines represents the k value and the horizontal grey discontinue line represent the %WS_max_ with have the 50% of its maximum valued. The results are the average of three experiments. The water segregation values corresponds to the difference between each experiment with spores and water segregated for the emulsion without the addition of spores at the same time (blank). Water segregated was determined by Karl-Fisher method.

In our case, fungal spores instead of biomass were used on assays since the latter had no demulsification effect and the former showed high hydrophobicity. The destabilization of the emulsions with different spore concentrations is shown in Fig 6.

For each crude oil it is observed that, for the most part, the segregation is nearly complete after 6 hours of incubation. The continue, discontinue line and discontinue-point lines represent least squares fits to a hyperbolic function from which the maximum water separation and half-time of destabilization (time required for separation to reach 50% of its maximum value) could be extracted.

The mechanism by which the fungal spores destabilize the emulsions is presumably due to a higher adsorption capacity of the spores at the interface in competition with other surface active species present in the emulsion such as asphaltenes, resins and NPE or the ingestion of NPE surfactant by the spores.

The half-life of the O/W emulsions diminished when the API density of the crude oils was decreased (Table 2). The shortest half-life was obtained with the emulsion prepared with extra-heavy crude oil, probably due to a higher resin and asphaltene content of this oil, which led to stronger interactions with the hydrophobic spores and thus to an enhanced ability to break the emulsion.

**Table 2:** Half-life of emulsions prepared with Mexican crude oils incubated with 2 g/L of spores of *Aspergillus* sp IMPMS7, including standard errors as calculated from the least square fitting procedure.

O/W Emulsion from	Half-life of emulsions (t_½_, h)	WS_max_^a^ (%)
Medium crude oil	6.12 ± 0.98	72.5
Heavy crude oil	4.40 ± 1.30	68.2
Extra-heavy crude oil	0.39 ± 0.17	65.2
Reference without spore	49.5 ± 0.27	60.8

^a^ Maximum content of water segregated

Interestingly, the O/W emulsion with medium crude oil presented the lowest separation of water with the addition of spores, followed by the extra-heavy and heavy crude oils, respectively, showing that destabilization of the crude oil emulsion is a very complex phenomenon that depends on the combination of several factors and not just on the crude viscosity.

**Fig 7:**
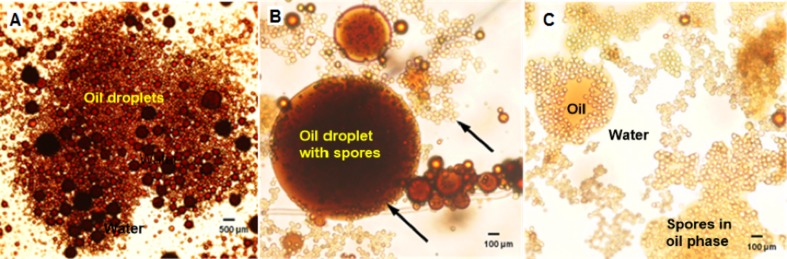
Microphotographs showing separation of water in the O/W emulsions, A) O/W emulsion from extra-heavy crude oil without spores, (B) O/W emulsion from heavy crude oil immediately after the addition of fungal spores (3.5 g/L). The spores are visible within both the crude oil and water phases (arrows), (c) After breaking of emulsion by spores. Magnification at 40 x.

The extra-heavy crude oil emulsion was observed after 2 minutes of the addition of fungal spores by optical microscopy (Fig 7). The images showed the fungal spores could penetrate the oil droplet, facilitating the coalescence of crude oil drops. The hydrophobic nature of fungal spores favors their incorporation into the crude oil phase as observed (Fig 7B). A comparison between the emulsion formed and broken by effect of the spores can be observed in micrographs 7A and 7B.

### Study of demulsification by fluorescence measurements

**Fig 8:**
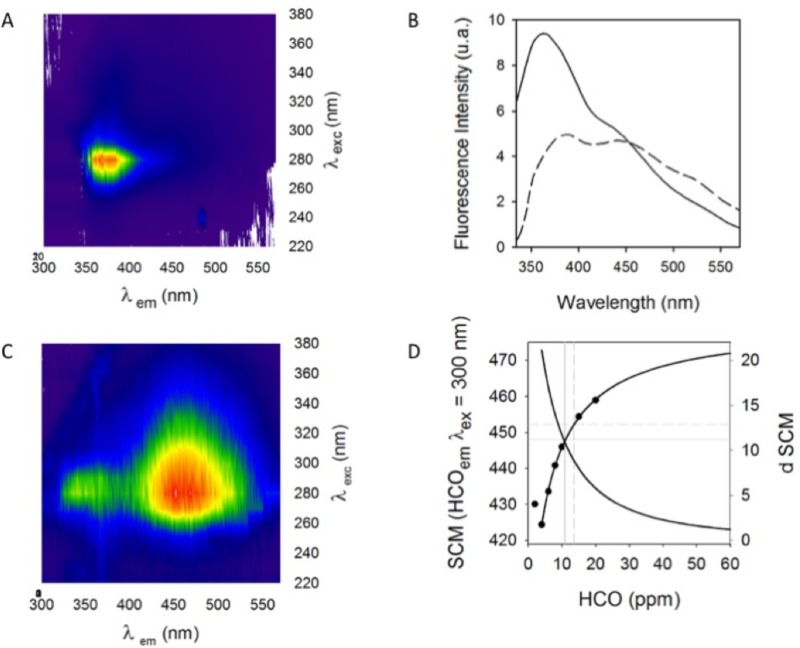
Behavior of the fluorescence intensity of the hydrocarbon emulsion in a water-surfactant dispersion. A) Spectrum 3D of the HCO emulsion in NPE solution at 2 ppm. B) Emission spectra (λex = 300 nm) of the emulsion dispersion at 2 ppm HCO (continuous line) and 20 ppm (dotted line). C) 3D Spectrum HCO NPE emulsion solution at a concentration of 20 ppm. D) Behavior of the emulsion dispersion in NPE solution depends on HCO aggregation.

The dispersion of a hydrocarbon emulsion in a NPE solution at 200 ppm shows an intrinsic fluorescence that is evident within an excitation length interval ranging from 260 to 369 nm and between an emission length interval ranging from 280 to 550 nm as observed in the tridimensional spectrum in Fig 8C, where the maximum hydrocarbon dispersion fluorescence at 2 ppm is observed in emissions close to 380 nm (Fig 8A); as the HCO concentration is increased up to 20 ppm, the displacement of the highest fluorescence intensity is observed near 460 nm (Fig 8C). This behavior pattern could be observed in a dimensional spectrum exciting at a wavelength of 300 nm, shown in Fig 6B, where the HCO emission spectrum at 2 ppm is represented with a continuous line, where the maximum emission is located at 360 nm, and it is diminished as the HCO concentration is diminished to 20 ppm (discontinuous line). The displacement phenomenon of the emission intensity can be described by the behavior pattern of the spectral center mass (SCM) curve as a function of the HCO concentration change (Fig 8D). The curve shown in Fig 8D describes a hyperbolic pattern that is associated with the function described in Eq 4 through which the aggregation constant (KS_aggr_) is calculated (vertical dotted line) and related to the dissociation or aggregation of hydrocarbon, which in this case is 14 ppm. In the case of the emulsion dispersion, the aggregation point (AP) was also calculated (vertical continuous gray line), which is the product of the intersection between the SCM curve and its derivative curve, and it is associated with the necessary hydrocarbon concentration to be aggregated, which in this case is 10.8 ppm. It is well known that the fluorometric technique permits the detection of small changes of the microenvironment surrounding fluorophore molecules such as those contained in crude oil [26].

The water separation kinetics from the emulsion prepared using heavy crude oil was studied by the fluorometric technique, determining the change in slope of aggregation and disaggregation of fluorescent compounds in the hydrocarbon during the breaking of the emulsion by the spore [3, 23, 27].

The fluorometry technique helps us to observe the phenomenon of aggregation and disintegration of the fluorescent compounds in the hydrocarbon whose origins are asphaltenes during the emulsion breaking by the spore; the aggregation change is interpreted by the slope change generated between the two emission peaks attributable to the aggregation and disaggregation zones of hydrocarbon, and are compared with the effect of a previously reported demulsifier chemical and bio-physical demulsification process [3].

**Fig 9:**
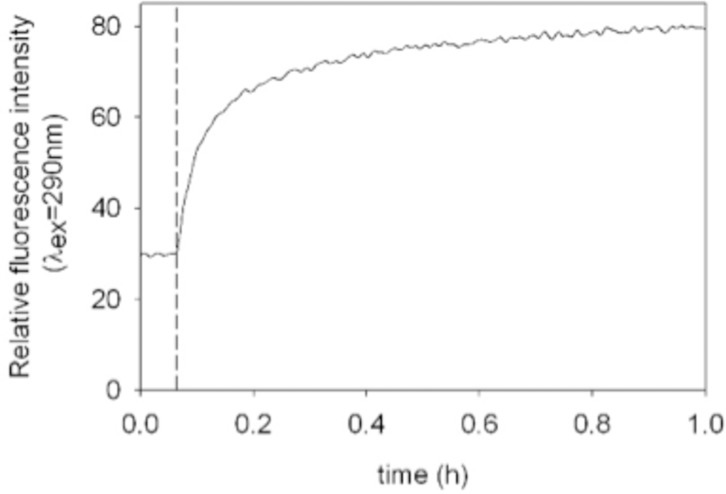
Fluorescence emission as a function of time for an O/W emulsion suspended in water and in presence of fungal spores. Conditions: λ_ex_ = 290 nm, λ_em_ = 340 nm; O/W emulsion concentration = 20 ppm; 1.0 g/L of fungal spores were added at t = 4 min (dashed line), temperature T = 34°C. Results shown are representative of 3 different experiments.

Preliminary studies were done in order to obtain fluorescence spectra of the O/W emulsions prepared with heavy crude oil (HCO) in the presence of spores at 1.0 g/L (Fig 9). The fluorescence emission of the O/W emulsion was maintained stable for 4 min until a noticeable intensity augmentation occurred when the fungal spores were added. During the first 4 min, the oil droplets quench the crude oil fluorescence emission until the addition of spores. Then, the increment in fluorescence emission may be associated with the breaking of the emulsified crude oil in the O/W emulsion and attributed to the dispersion of the fluorophore molecules present in the crude oil, such as polycyclic aromatic hydrocarbons (PAHs), asphaltenes and resins [28, 29].

Thus, by observing the emission spectra of a heavy crude oil and its emulsion (both in the presence and absence of fungal spores), the effect of the spores on emulsion breaking was studied (Fig 7). The micro environmental changes around crude oil molecules being emulsified or in contact with fungal spores may be tracked by spectral analysis as described by Murillo-Hernández et al. [22]. They described two general association states of crude oil molecules: non-aggregated or dispersed molecules that emit light at higher energy (lower wavelength) than the aggregated molecules, which emit in at a higher wavelength range from 300 to 420 nm.

**Fig 10:**
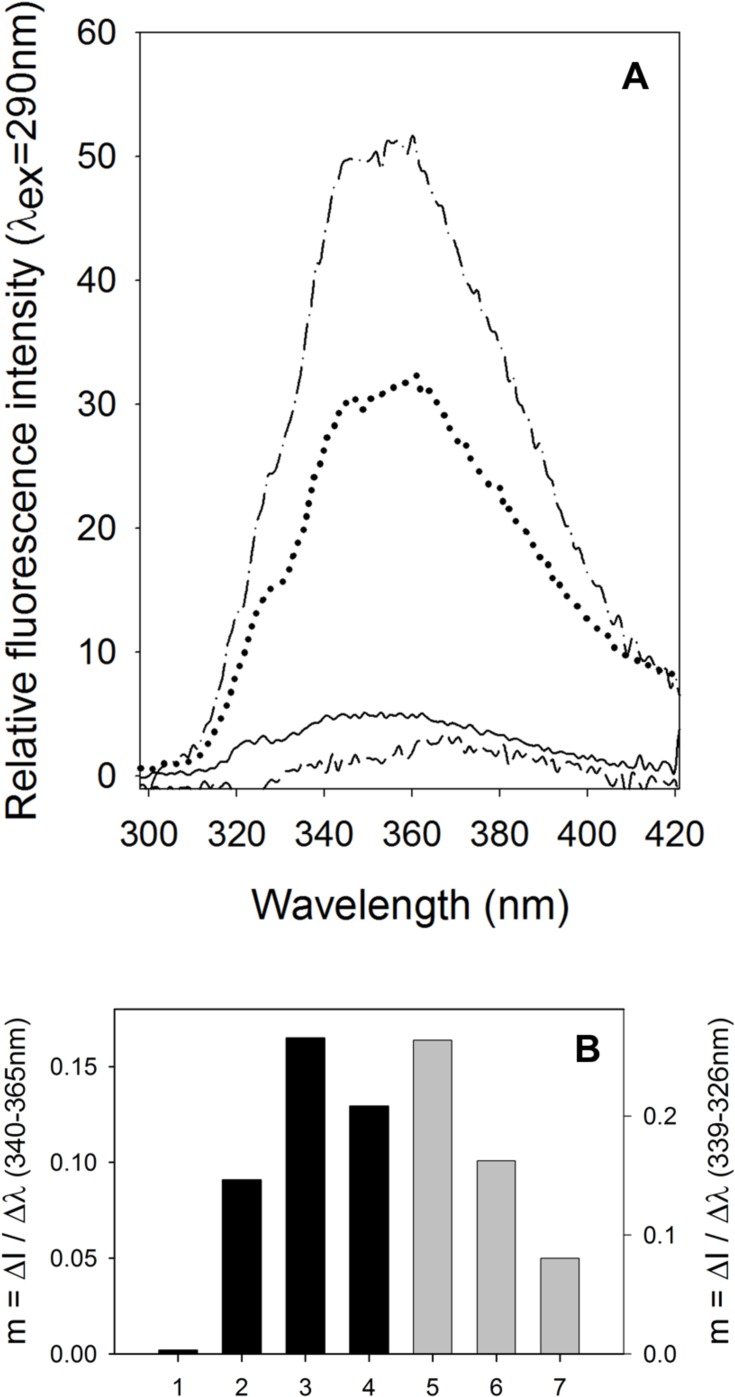
Dispersed aqueous solutions showing A) the emission spectra of crude oil (—), crude oil with fungal spores (–––), O/W emulsion (● ● ●) and O/W emulsion with fungal spores (–● – ● –); and B) the I_365_/I_340_ ratio of crude oil (1), crude oil with fungal spores (2), O/W emulsion (3), O/W emulsion with fungal spore (4), the I_339_/I_326_ ratio of O/W emulsion (5), O/W emulsion broken in presence of demulsifying agent (6) and O/W emulsion treated with MW irradiation at 60°C (7), (5, 6 y 7) data in ref. [3]. Conditions of fluorometric studies: λex = 290 nm, T = 25°C.

The spectrum of the neat crude oil dispersed in water (200 ppm) showed a symmetric behavior indicating that both non-aggregated and aggregated fluorophore molecules contribute equally to the low emission fluorescence of the crude oil (solid line). Such a low fluorescence emission is due to the well-known self-quenching effect that diminishes the emission intensity. This has been described by Imhof et al. [29] using a model of fluorescein dyed colloidal silica spheres, which at high concentrations exhibit self-quenching. The spectrum changed in the presence of fungal spores; two dominant peaks appeared at 340 and 365 nm (Fig 10A, dashed line). The intensity ratio of the two peaks, I_365_/I_340_, was increased relative to the pure crude oil indicating an increased presence of aggregated crude oil molecules (Fig 10B). This fact can be attributed to the hydrophobic character of the spores which cause the aggregation of polar molecules present in the crude oil.

In the case of the O/W emulsion, a considerable increment in fluorescence emission (Fig 10A, dotted line) was observed due to the homogenous dispersion of oil drops in the aqueous bulk that diminished the quenching effect. When the spores were added to the emulsion, the fluorescence emission increased still further, corresponding to the dynamic interaction between the spores and crude oil droplets that further diminished the quenching effect. With regard to the I_365_/I_340_ ratio, its value fell with respect to the spore-free emulsion, indicating an increased presence of aggregated molecules (Fig 10B).

According to the data of Fig 10B, the chemical demulsifier employed appears to be better than the spores; however the waits are significantly advantageous due to their low cost and low toxicity compared to the nitrogen de-emulsifiers.

Furthermore, from the fluorescence as a function of wavelength l(λ), the values of the Δl/Δλ gradients were also plotted (Fig 10B). These are an additional tool to study the microenvironment between the emulsion drops and the interaction with the spores. If the emulsion breaks, the slope should be similar to the behavior of the hydrocarbon in the presence of the spores, since the spores contribute to the change in the oil dispersion. The hydrocarbon slope in the seawater (bar 1) is 1.83x10^-3^, which increases approximately 50-fold in the presence of the spores (bar 2). The dispersion slope of the emulsion in seawater (bar 3) is 0.1649, which decreases to 0.12954 when the spores are added (bar 4). This decrease tends towards the value of the heavy hydrocarbon in the presence of the spores, indicating that there is different interaction between the hydrocarbon and the spores compared to between the hydrocarbon and seawater alone. This is confirmed by the micrographs in Fig 5.

In order to clarify further the phenomena involved, two more, distinct emulsion breaking experiments were performed using of an ammonium-based demulsifying agent to break the emulsion (Fig 8B, bar 6) and heating the emulsion (80°C) with microwave (MW) irradiation (Fig 10B, bar 8). Again, a similar trend to the one observed with the use of fungal spores was obtained, i.e. that the I_339_/I_326_ ratio diminished for both types of treatment, indicating an increased presence of aggregated molecules [3]. The calculated slope when the chemical demulsifier is employed (bar 6) turns out to be 38% less than the slope of the emulsion alone (bar 5). When the emulsion is subjected to a physical procedure such as breaking by microwaves, the obtained slope is 70% less than the slope of the emulsion alone (bar 7), indicating a higher demulsification degree. A greater demulsifying effectiveness of microwaves may be due to the dielectric heating and the friction between molecules and the increase in the ionic collision rate generated by alignment and relaxation of the dipoles under the electromagnetic field [3]. On the other hand, the use of chemical demulsifier agents or the fungal spores needs close contact between the oil drops and the demulsifying agents to favor interaction and further coalescence.

This determination of the slopes offers an alternative method to analyze the change in the emission spectra when the bathochromic shift is not clear. The other option mentioned above is through the calculation of the spectral center of mass (SCM) described by Murillo-Hernández et al. [22]. They observed an SCM value of 361.68 nm when using the spores, which is similar to that of the hydrocarbon, 358.99 nm. This hypsochromic shift is indicative of an emulsion breaking.

According to this study, the demulsification process occurs immediately–this fact was observable through an instant change in emission fluorescence.

## Conclusions

Fungal cultivations were isolated from sediments contaminated with petroleum hydrocarbons. The spores of *Aspergilllus sp*. IMPMS7 showed a high hydrophobicity of (89.3 ± 1.9) %. These spores were able to break emulsions of medium, heavy and extra-heavy crude oil in water previously emulsified by means of a nonylphenyl ethoxylated surfactant.

As observed through an optical microscope, the emulsions created with the extra-heavy crude oils formed droplets, which in the presence of fungal spores, collided and promoted a fast emulsion breaking. They also produced an agglomeration of spores around the surface oil slick, provoking the breaking (half-life decreases sharply), suggesting that the hydrophobic fungal spores had a greater affinity for oil components which surrounding the oil droplets.

The demulsification process was studied by fluorescence spectroscopy which showed that the process begins immediately. This was in accordance with the observations by optical microscopy and of the water separation.
